# Less may be more: nodal treatment in neck positive head neck cancer patients

**DOI:** 10.1007/s00405-015-3634-5

**Published:** 2015-04-29

**Authors:** Gabriela Studer, Gerhard F. Huber, Edna Holz, Christoph Glanzmann

**Affiliations:** Department of Radiation Oncology, University Hospital Zurich, Raemistrasse 100, 8091, Zurich, Switzerland; Department of Otorhinolaryngology, Head and Neck Surgery, University Hospital Zurich, Zurich, Switzerland

**Keywords:** Planned neck dissection, PND, Elective nodal volume and radiation, Isolated nodal failure, Nodal IMRT

## Abstract

Ongoing debates about the need and extent of planned neck dissection (PND), and required nodal radiation doses volumes lead to this evaluation. Aim was to assess nodal control after definitive intensity modulated radiation therapy (IMRT ± systemic therapy) followed by PND in our head neck cancer cohort with advanced nodal disease. Between 01/2005 and 12/2013, 99 squamous cell cancer HNC patients with pre-therapeutic nodal metastasis ≥3 cm were treated with definitive IMRT followed by PND. In addition, outcome in 103 patients with nodal relapse after IMRT and observation only (no-PND cohort) were analyzed. Prior to PND, PET–CT, fine needle aspirations, ultrasound and palpation were assessed regarding its predictive value. Patterns of nodal relapse were assessed in patients with isolated neck failure after definitive IMRT alone. 70/99 (70 %) PND specimens showed histopathological complete response (hCR), which translated into statistically significantly superior survival compared with partial response (hPR) with 4-year overall survival, disease specific survival and nodal control rates of 90/83/96 vs 67/60/78 % (*p* = 0.002/0.001/0.003). 1/99 patient developed isolated subsequent nodal disease. 64/2147 removed nodes contained viable tumor (3 %). Predictive information of the performed diagnostic investigations was not reliable. 17/70 hCR patients showed true negative findings in available three to four investigations (0/29 hPR). 27/103 no-PND patients developed isolated neck disease (26 %) with successful salvage in 21/24 [88 %, or 21/27 (78 %)]. Nearly all failures occurred in the prior nodal gross tumor volume area. A more restrictive approach regarding PND and/or nodal IMRT dose-volumes may be justified.

## Introduction

In several publications, low isolated neck recurrence rates after irradiation (±chemotherapy) of ~<5 % is reported [1–6]. Main question is, if full radiation dose aiming a biological dose equivalent of ~70 Gy to nodal metastases with more or less extended elective irradiation of the lymphatic pathways, combined with systemic therapy in most cases, followed by planned neck dissection (PND), may or not be needed [1, 7, 8]. According to our internal guidelines, PND in all patients with initial nodal disease >3 cm referred for definitive intensity modulated radiation therapy (IMRT ± systemic therapy) was provided since 2005.

Aim was to assess the outcome in this cohort. We evaluated the rate of residual disease in PND specimens and predictive values for complete remission of positron emission tomography–computed tomography (PET–CT), fine needle aspiration (FNA), ultrasound (US) and clinical palpation of the neck prior to PND.

In addition, the pattern of nodal relapses was assessed in cN+ (any nodal size) patients with neck failure after definitive IMRT followed by observation only (no-PND cohort). Hypothesis was that de-intensivation of neck treatment may be an option.

## Methods

All patients were identified from a prospective data base. Ethical approval for IMRT cohort analysis is available. Histopathological disease control in the PND cohort, nodal failure patterns in PND and no-PND patients (localization in the prior nodal gross tumor volume (GTV) inside the boost planning target volume (PTV), vs inside the intermediate or elective PTV, vs outside of any treatment volumes, respectively), and rates of isolated nodal failure and its successful salvage in both cohorts was assessed.

### Patients

Between 01/2005 and 12/2013, 99 HNC patients with cervical lymph node metastases ≥3 cm were treated with definitive IMRT ± systemic therapy followed by PND. Patient and tumor characteristics are shown in Tables 1, 2.

**Table 1:** Cohort characteristics

Parameters	PND cohort (*N* = 99)	No-PND cohort with nodal failure (*N* = 103)
Gender (f:m)	21:78 (1:3.7)	1:4.1
Mean age at initial treatment (range)	61 (41–83)	61 (27–91)
Mean follow-up, (months/range)	49 (6–107)	23 (2–100)
Squamous cell carcinoma histology	99 (100 %)	103 (100 %)
Diagnosis		
Mesopharynx	69 (70 %)	53 (52 %)
Hypopharynx	19 (19 %)	21 (21 %)
Larynx	9 (9 %)	11 (10 %)
Unknown primary	2 (2 %)	1 (1 %)
Oral cavity	0	13 (12 %)
Nasopharynx	0	4 (4 %)
TN stages		
T 1/2/3/4	18/42/14/25/0	7/26/20/41/9
N 0/1/2a/2b/2c/3/recurrence	0/2/9/48/32/8/0	0/10/3/37/42/5/6

**Table 2:** Disease characteristics

Disease parameters	All PND (*n* = 99)	hCR PND (*n* = 70)	hPR PND (*n* = 29)	*p* value
Largest node diameter	45.5 mm (30–86)	44.2 mm	48.6 mm	NS
Volume of the largest node	21.1 cc (6–131)	20.5 cc	22.4 cc	NS
Total initial nodal volume	31.5 cc (6–131)	29.3 cc	37.5 cc	NS
T 1/2-3/4	18/56/25	11/39/20	7/18/5	NS
N 1-2b/2c/3	59/32/8	42/23/5	17/9/3	NS
Diagnosis				
Hypopharynx	19	9	10	NS
Larynx	8	5	3	na
Oropharynx	72	56	16	NS

In addition, our no-PND cohort with proven nodal relapse/progress following definitive IMRT (103 of 718 IMRT patients (14 %)) treated between 02/2002 and 12/2013 was analyzed, Table 1. Reasons for no previous PND in those patients were: treatment prior to 2005 (*n* = 13), cN+ <3 cm (*n* = 31), early salvage surgery for clinical or radiological high suspicion for/proven loco-regional persistence (*n* = 41), or patients’ preference or comorbidity or age (19).

### IMRT

Treatment plans were calculated by the Varian Treatment Planning System (Eclipse^®^ External Beam Planning System, Version 7.3.10 and PRO 8.9, AAA 8.9, Varian Medical Systems). Simultaneously integrated boost (SIB)–IMRT was performed using the following schedules (for more details see former publications [9])

Definitive IMRT

- SIB2.00: Daily dose of 2.00 Gy (PTV1)/1.70 Gy (PTV2)/1.54 Gy (PTV3) to a total dose of 70.00 Gy (5 fractions/week).
- SIB2.11: Daily dose 2.11 Gy (PTV1)/1.80 Gy (PTV2)/1.64 Gy (PTV3) to a total dose of 69.60 Gy (5 fractions/week).
- SIB2.2: Daily dose 2.2 Gy (PTV1)/2.0 Gy (PTV2)/1.64 Gy (PTV3) to a total dose of 66.0 Gy (5 fractions/week).

The elective nodal treatment volume was mostly covered by 54 Gy in 33–35 fractions; nodal metastases were treated with 70 Gy to the nodal high-dose planning target volume [PTV, 8–12 (−15) mm margin around nodal gross tumor volumes].

### Concomitant systemic therapy

Cisplatin was given in weekly doses of 40 mg/m^2^ at 1 day a week. Since 04/2006, cetuximab (400 mg/m^2^ loading dose, followed by 250 mg/m^2^ at 1 day a week) was used in patients with contra-indications for concomitant standard cisplatin chemotherapy. 87 and 82 % of the PND and no-PND cohort underwent concomitant systemic treatment, respectively.

### Follow-up (FU) after IMRT

3–6 weeks after completion of IMRT, all patients were regularly seen in our joint clinic at the Department of Head and Neck or Maxillofacial Surgery. Institutional standards for patient assessment included physical examination with additional flexible fiber-optic endoscopy approximately every 2 months in the first year of follow-up, every 3 months in the second to third year, and every 6 months in the fourth to fifth year. Most patients completed FU after 5 years of relapse free observation. Our interdisciplinary in-house guidelines recommend an elective neck dissection in patients with initial nodal metastasis >3 cm since 2005. All patients with performed elective PND in the time interval between 3 and 18 weeks were included in this analysis. Patients with proven persistent or early progressive disease based on clinical examination and/or imaging and/or histopathological prove were not included in this PND evaluation (*n* = 7). Rates of ultrasound, PET–CT, FNA performed are listed in Table 3.

**Table 3:** Predictive value of PET–CT and FNP in the assessed cohort

	All END (*N* = 99)	hCR END (*n* = 70)	hPR END (*n* = 29)
PET-CT			
Available	68/99 (69 %)	50/70 (71 %)	18/29 (62 %)
Correct prediction	49/68 (72 %)	CN: 43/50 (86 %)	CP: 6/18 (33 %)
FNA			
Available	50/99 (50 %)	35/70 (50 %)	15/29 (50 %)
Correct prediction	33/50 (66 %)	CN: 27/35 (77 %)	CP: 6/15 (40 %)
PET-CT + FNA available	39/99 (39 %)	25/70 (36 %)	14/29 (48 %)
Both predictions correct	19/39 (49 %)	CN: 15/25 (60 %)	CP: 4/14 (29 %)
No PET-CT and no FNA available	21/99 (21 %)	13/70 (19 %)	8/29 (28 %)
Ultrasound			
Available	80/99 (81 %)	66/70 (94 %)	24/29 (83 %)
Correct prediction	31/99 (31 %)	CN: 8/66 (12 %)	CP: 23/24 (96 %)
Cervical palpation			
Information available	99/99 (100 %)	70/70 (100 %)	29/29 (100 %)
Correct prediction	61/99 (62 %)	CN: 35/70 (50 %)	CP: 26/29 (90 %)
No positive investigations	17/99 (17 %)	17/70 (24 %)	Zero (0 %)

*PND* planned neck dissection, *hCR* histological complete remission, *hPR* histological partial remission, *CN* correct negative, *CP* correct positive

### Statistics

Statistical calculations were performed using the statistics program implemented in StatView^®^ (version 4.5; SAS Institute, Cary, NC). Univariate analyses were performed with a Cox proportional hazards regression model in StatView^®^. Actuarial survival data were calculated using Kaplan–Meier curves and log-rank tests implemented in StatView^®^. *p* values <0.05 were considered statistically significant.

## Results

### Survival

PND cohort: when last time seen, 74 % were alive with no evidence of disease, 7 % were alive with disease, 11 % have died of disease, and 8 % have inter-currently died. In 65 %, PND was performed 3–8 weeks after IMRT completion, in 23 %, in the interval between >8 and 12 weeks, in 11 %, the interval was >12–17 weeks.

no-PND cohort: 103/718 (14 %) of all patients with nodal relapse following definitive IMRT ± systemic therapy and observation only were assessed. In 39/103, persistent nodal disease was diagnosed (persistence: 0–6 months post IMRT). The 4-year OAS was 73 %, comparable to the PND cohort.

### PND specimens

**Fig. 1:**
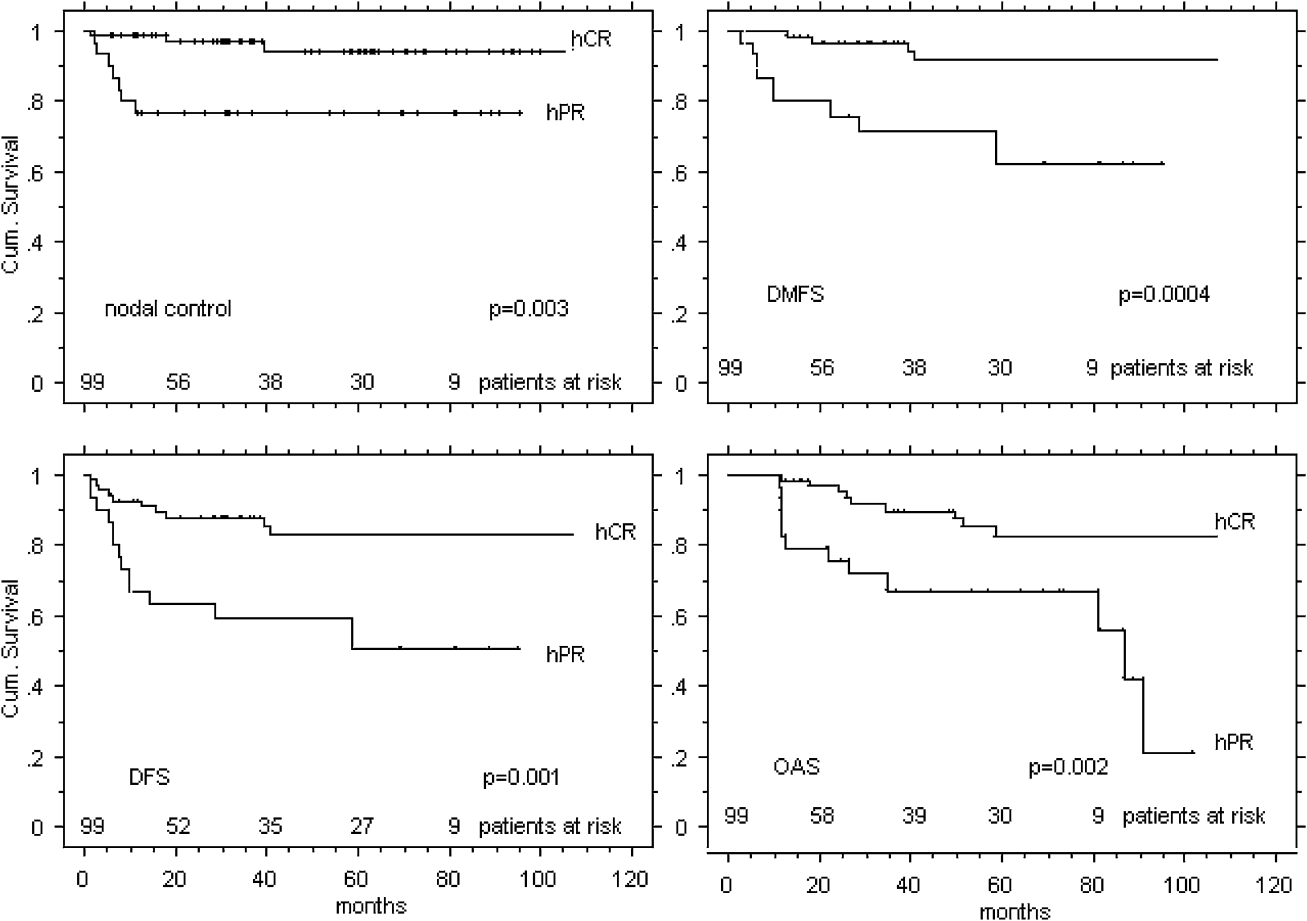
PND cohort: disease control

In 70/99 (70 %) patients, PND specimens showed histopathological complete response (hCR), and in 29/99 (30 %) partial response (hPR), Table 2. Disease control rates are listed in Table 4 and Fig. 1. 14/29 (48 %) hPR patients and 9/70 (13 %) hCR patients experienced subsequent distant and/or local and/or nodal failure.

**Table 4:** Disease control

4-Year outcome parameters	hCR PND (*n* = 70) (%)	hPR PND (*n* = 29) (%)	*p* value
Local control rate	91	88	0.56
Nodal control rate	96	78	0.003
Distant metastastis free survival	95	71	0.0004
Disease specific survival	83	60	0.001
Overall survival	90	67	0.002

In 16/29 hPR patients, the specimen contained one single node with viable cells, in the remaining 13 patients, 2 (*n* = 6), 3 (*n* = 2), 4 (*n* = 3), 6 (*n* = 1) and 11 nodes (*n* = 1) were found. 8/13 (62 %) developed distant and/or local and/or nodal failure in the following 2–6 months, which occurred in 6/16 (38 %) patients with only 1 positive lymph node. 64/2124 removed nodes contained viable tumor (3 %). 18/99 patients (18 %) underwent bilateral PND (viable tumor in 4/18 (4 %); in all 4 patients both sides were affected (8 hPR specimens out of totally 117 neck dissection specimens, 7 %). The following levels were removed in 81 patients with unilateral PND: level ll (2), level lll (1), levels ll–lll (7), levels l–lll (2), levels ll–lV (53), levels l–lV (12), levels ll–V (4).

### Prediction of hPR in PND specimens

TN stage, nodal diameter or gross tumor volume were not predictive for hPR. Table 3 shows results of the assessed investigations. In 16/29 patients (55 %), a very low viable tumor cell load was described in the histopathological report (‘minimal viable residuals’ or ‘few viable cells left’); in 10 patients viable tumor areas in the range of one to several millimeters were reported, in only 3 patients (10 % of hPR, 3 % of the entire cohort), substantial viable tumor persistence was found like soft tissue metastasis or several necrotic lymph nodes with extra-capsular extension. In 2 of these 3 patients, pre PND FNA and PET–CT were positive. 17/70 hCR patients (24 %) showed no signs of disease in all (3–4 each) performed investigations; in the hPR cohort, all patients presented with positive/highly suspicious findings in at least one of their 1–4 investigations.

hCR translated in statistically significantly superior outcome compared to hPR, Table 4 and Fig. 1. All outcome parameters but local control were statistically significantly different in favor of hCR.

### Isolated nodal failure

**Fig. 2:**
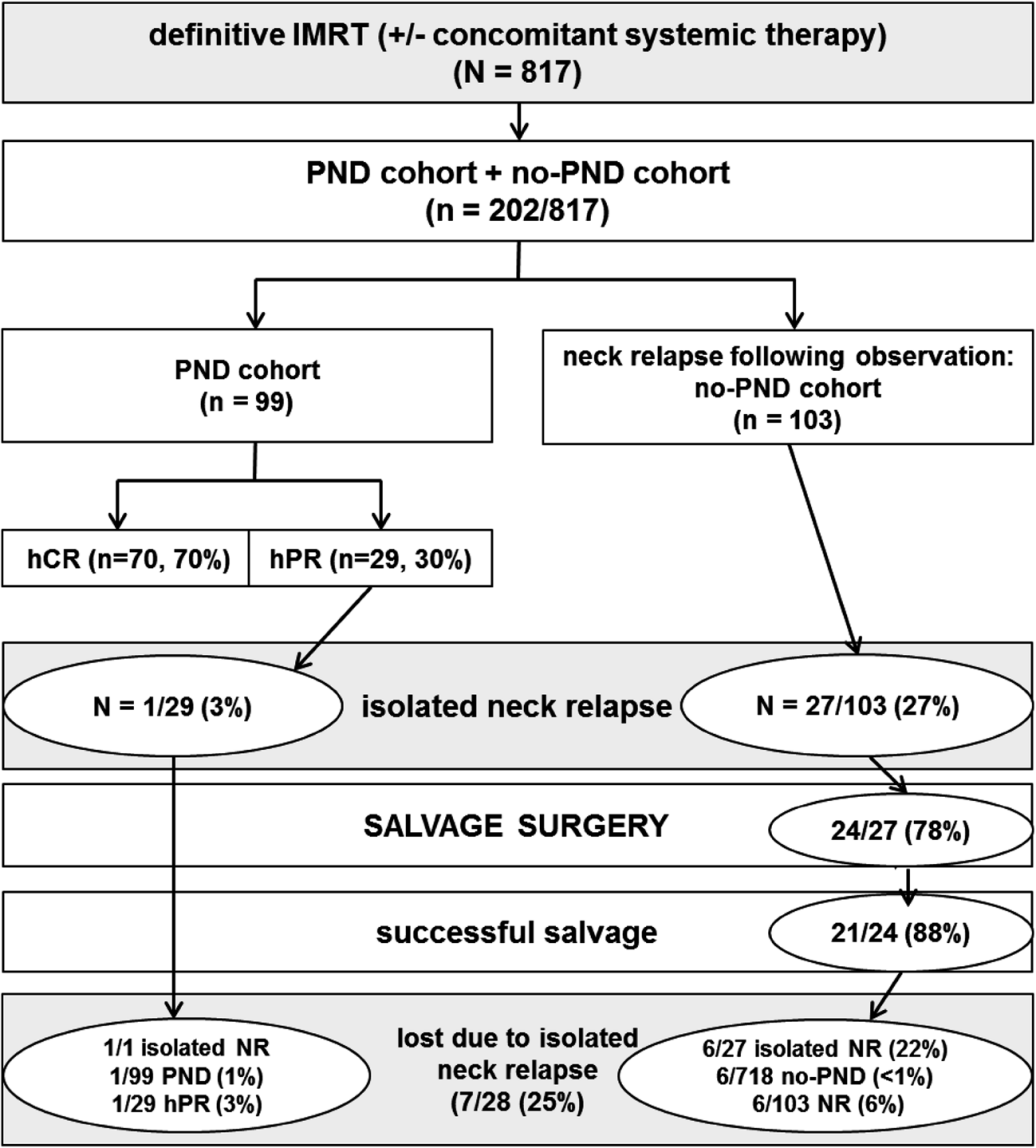
Outcome in patients with isolated nodal disease in the two assessed cohorts

Isolated nodal failure occurred in only 1/29 (3 %) patient subsequently to PND (1/14 in the hPR subgroup). In the no-PND cohort, 27/103 patients (26, or 4 % of the entire IMRT cohort) presented with an isolated nodal relapse. In 24/27 (89 %), salvage surgery was performed, in 21/24 (88 %) successfully: 6/27 (22 %) of the patients with isolated nodal relapse have been lost, or 0.8 % of all IMRT patients who underwent no PND (6/718). Figure 2 gives an overview about the two analyzed cohorts with respect to outcome in patients with isolated nodal disease.

### Site of nodal persistence

PND cohort: In 28/29 patients with a residuum in the hPR specimen, residual disease was identified at the anatomical site of the initially positive nodes; in 5 patients with initially advanced disease and substantial viable residuals, disease was found at the original nodal gross tumor volume as well as invading the environment.

no-PND cohort: 91 % (94/103) of the no-PND cohort were diagnosed in the high-dose PTV where the initial nodal GTV was located, in 3 % nodal relapse developed in the intermediate or elective dose PTVs, in 6 % out of the radiation PTVs. 26 of 27 patients with isolated nodal disease developed nodal failure in the high-dose area in/close to the prior GTV, 1/27 developed in the elective PTV.

## Discussion

Limitations of the present evaluation are––beside the retrospective evaluation of the no-PND cohort––the aspect that not all patients underwent FNA and/or PET-CT and/or ultrasound prior to PND, as the indication for PND was previously decided (‘planned’) based on the nodal metastasis diameter.

An early analysis of failure patterns after definitive IMRT of our first 280 patients [10] is included in this recent analysis, with comparable nodal failure rate (11 vs 14 %).

In brief, we found

- 30 % of PND specimens in patients with nodal metastasis >3 cm containing residual histopathological disease
- 3 % of resected PND lymph nodes containing viable tumor
- all hPR patients presented with 1–4 positive pre-operative examination results (PET–CT, US, FNA, palpation); 17 of the hCR group showed correct negative findings in their 3–4 available examinations; for this situation, the estimated risk to miss residual disease by observation only would be ~18 % (acc. to the statistical ‘rule of three’ to estimate the probability of adverse events in small sample sizes with few events, giving the upper limit of the 95 % confidence interval of the probability: 3/17)
- isolated neck relapse was diagnosed in 1 % subsequently to PND, in 27 % of our no-PND neck relapse cohort, of which ~80 % were successfully salvaged
- >95 % of all nodal failures/residua were found at the site of prior nodal GTV.

Comparison with published data:

### PND specimen

Our findings match well with recent published literature. Response rates to radiation are reported to some degree influenced by the time gap between PND and completion of radiation; however, there is considerable congruence with published results from other centers. Goguen et al. [11] reported a clinically complete response rate of 75 % (61–85 %), Dautrement et al. [12] found 68 %, Cannady et al. [3] 65 % hCR in 241 patients all with cN2–3 disease. Ganly et al. [13] found 19/56 patients (33 %) with viable tumor in their specimen of a comprehensive neck dissection either as a planned or salvage procedure. Hamoir et al. [7] conducted a recent literature review on the role of neck dissection following chemo-radiation, and summarized the reported rates of viable nodal tumor cells as follows: 40 % (20–68 %) in cases of clinical partial nodal response, and 20 % (0–39 %) in cases of clinical complete response, noting that the ranges may be explained by factors like the interval between completion of radiation and END, or the criteria to establish the viability of tumor cells. Most recently, Huang et al. [14] reported on 291 patients treated with PND after radiation alone or for radiologically diagnosed residual disease, finding 28 % with residual viable tumor foci in the specimen.

### Predictive parameters for hPR

FNA and PET–CT were limitedly reliable to predict hCR/hPR, which is expectable, considering that 55 % of the specimens in our cohort contained only few viable cells. In addition, in 35 % viable tumor areas of several millimeters to larger areas of the affected mostly substantially altered and destroyed lymph nodes with no extra-capsular extension were stated. This finding confirms review results published by Hamoir et al. [7], who summarized reported data of a poor correlation between physical examination plus CT and pathological diagnosis (negative predictive value 33–44 %). With respect to FDG–PET–CTs, we are faced with conflicting results from retrospective studies (different time intervals from radiation, as one of several potential reasons) [7].

Hypopharynx carcinoma was reported baring a higher risk as compared to other primary sites [1]. This was also found in our cohort with however too small sample size to rely on (10/19 hypopharynx cancer patients with hPR).

### Survival benefit for hCR patients

Approximately 20 % survival benefit was similarly observed by Soltys et al. [15] in patients who underwent neck dissection for clinically partial response, with 75 % (hCR) vs 42 % (hPR) 5-year disease free survival. Ganly et al. [13] reported 5-year overall survival rates of 93 vs 49 % in favor of the hCR subgroup, Lango et al. [16] found recurrence free survival rates of 85 vs 37 % for negative vs positive pathology.

Half of our patients with hPR subsequently developed loco-regional and/or distant failure (15/29). Huang et al. [14] similarly found 33 subsequent failures in 70 patients with positive PND. The ultimate cure rate of patients with viable nodal tumor is reported ~30–50 % [13, 14, 16], ~66 % in the own cohort.

### Location of nodal residuum or relapse in the prior GTV area

As a consequence of the known fact of most loco-regional failures following definitive IMRT occurring in-field (inside the boost PTV) in the area of prior primary and nodal gross disease [10, 17–19], the recent trend in head neck cancer IMRT is to generally reduce boost PTV margins around the gross tumor volume (GTV, e.g. EORTC trial 1219, also e-book N.Y. Lee, J.J. Lu, *Target Volume Delineation and Field Setup*, 317 DOI 10.1007/978-3-642-28860-9, 2013) [17]. The anatomical patterns of neck failure and persistence in our no-PND and hPR subgroup confirmed this fact. Similarly, Garden et al. [5] found only 2 % (12/776) of definitive IMRT (±chemotherapy) patients with loco-regional recurrences outside the high-dose target volumes after a median follow-up of 54 months. 3 % (64/2124) of all dissected nodes in our PND cohort contained residual viable tumor, which raises again the ongoing debate on the required extent of neck dissection. Robbins et al. [20] conducted a prospective study on 84 patients who underwent neck dissection (radical or modified radical neck dissection vs selective neck dissection vs super-selective neck dissection) for bulky nodal disease, resulting in non-significantly different OAS and distant metastasis free survival.

### Isolated nodal relapse

Isolated nodal relapse is constantly reported being low subsequent to PND as well as after radio-chemotherapy alone. This was 1 % in the own PND cohort.

The Trans Tasman Radiation Oncology Group (TROG) 98.02 Study was determined to assess the incidence of isolated nodal failure after radiation in 102 patients with N2/3 disease and found zero isolated nodal failure without a planned neck dissection, but 29/102 patients with distant spread [21]. Garden et al. [5] assessed patterns of disease recurrence following definitive IMRT in 776 oropharyngeal cancer patients treated between 2000 and 2007 and found 3 % isolated nodal recurrences (N0: 88, N1/x: 140, N2a/b: 370, N2c: 122, N3: 56). Similarly, many other centers reported low rates <5 % [1–4, 6, 14, 20].

In our no-PND cohorts this was 27 %, of which ~80 % were cured by salvage surgery. Adams et al. [23] reported isolated nodal failure of 6 % in 33 irradiated patients with N3 status with neck dissection in 4 cases. The authors concluded that the predominant pattern of relapse is metastatic (30 %), and therefore intensification of systemic therapy rather than loco-regional therapy should be explored. Alternatively, reducing the radiation dose to the nodal GTV and the elective volumes may be another option to decrease treatment intensity without compromising oncological outcome.

### Salvage neck dissection

In 75 % of our small isolated neck relapse cohort (*n* = 28, Fig. 2), successful salvage surgery was achieved. Similarly, Deschamps et al. [23] reported from a tumor registry based study on 224 (35 %) patients with disease recurrences out of 1291 who were irradiated between 1998 and 2007. 47/224 (21 %) patients presented with isolated neck recurrence. Lee and her team assessed 260 nodally positive head neck cancer patients treated with definitive radiation (−chemotherapy) who all underwent observation only after a negative PET–CT scan 6–24 weeks post-radiation. 4/260 patients (2.3 %) developed isolated neck relapse, of which 3/4 underwent successful salvage neck dissection. In 4/16 who underwent post-radiation ND because of negative PET–CT but clinical suspicion ± positive CT or MRI, residual nodal disease was found; all patients achieved regional control [6].

## Conclusion

Considering the facts of most neck relapses occurring in the prior GTV, isolated nodal failure counting for ~25 % of nodal relapses after IMRT only, of which ~80 % can be salvaged, reduction of the radiation dose to nodal metastases provided for PND, and/or a more restrictive approach to PND may be suggested.
